# Automated Epileptic Seizure Detection Based on Wearable ECG and PPG in a Hospital Environment

**DOI:** 10.3390/s17102338

**Published:** 2017-10-13

**Authors:** Kaat Vandecasteele, Thomas De Cooman, Ying Gu, Evy Cleeren, Kasper Claes, Wim Van Paesschen, Sabine Van Huffel, Borbála Hunyadi

**Affiliations:** 1 KU Leuven, Department of Electrical Engineering (ESAT), STADIUS Center for Dynamical Systems, Signal Processing and Data Analytics, Leuven 3001, Belgium; thomas.decooman@kuleuven.be (T.D.C.); ying.gu@kuleuven.be (Y.G.); sabine.vanhuffel@esat.kuleuven.be (S.V.H.); bori.hunyadi@esat.kuleuven.be (B.H.); 2 imec, Leuven 3001, Belgium; 3 KU Leuven, University Hospital, Department of Neurosciences, Leuven 3000, Belgium; evy.cleeren@uzleuven.be (E.C.); wim.vanpaesschen@uzleuven.be (W.V.P.); 4 UCB, Brussels 1070, Belgium; Kasper.Claes@ucb.com

**Keywords:** epilepsy, seizure detection, home monitoring, long-term monitoring, wearables, photoplethysmography, electrocardiography

## Abstract

Electrocardiography has added value to automatically detect seizures in temporal lobe epilepsy (TLE) patients. The wired hospital system is not suited for a long-term seizure detection system at home. To address this need, the performance of two wearable devices, based on electrocardiography (ECG) and photoplethysmography (PPG), are compared with hospital ECG using an existing seizure detection algorithm. This algorithm classifies the seizures on the basis of heart rate features, extracted from the heart rate increase. The algorithm was applied to recordings of 11 patients in a hospital setting with 701 h capturing 47 (fronto-)temporal lobe seizures. The sensitivities of the hospital system, the wearable ECG device and the wearable PPG device were respectively 57%, 70% and 32%, with corresponding false alarms per hour of 1.92, 2.11 and 1.80. Whereas seizure detection performance using the wrist-worn PPG device was considerably lower, the performance using the wearable ECG is proven to be similar to that of the hospital ECG.

## 1. Introduction

Epilepsy is one of the most common neurological disorders, and it affects almost 1% of the population worldwide [1]. Anti-epileptic drugs provide only adequate treatment for about 70% of epilepsy patients [2]. After diagnosis in the hospital, one needs a follow-up of the disease and evaluation of the treatment. This follow-up requires a seizure logging system that is functional in a daily life environment outside the hospital, such as a seizure diary. A seizure diary, kept by patients or their families, is unfortunately not reliable [3].

To log seizures in an objective way, there is a need for an automatic seizure detection device, which records biomedical signals of the patient during daily life. On the basis of these signals, a computer-based algorithm using signal processing and machine learning can be used to automatically detect the seizures. With this information, an electronic diary can be generated. Ideally, the device will be worn continuously day and night, so it is important that the device is wearable and comfortable. Furthermore, it should be as concealable as possible to reduce stigmatization. However, it is important to have high data quality in order to have a reliable seizure detection. This trade-off between patients’ comfort and data quality should be investigated for different wearable devices.

Nowadays, the golden standard for recording epileptic seizures in the hospital is based on video-electroencephalography (EEG). This type of EEG recording requires wet electrodes on the scalp, which is uncomfortable for the patient, and a trained nurse is needed to position them. Furthermore, the detection is based on manual human assessment. As a result, a trained EEG analyst is needed to analyze the EEG, which is time consuming. Therefore, EEG is currently not suitable for an automated wearable long-term seizure detection system at home [4].

Other biomedical signals used to detect epileptic seizures include accelerometry (ACC), electromyography (EMG), galvanic skin response and electrocardiography (ECG). The most suitable modality or combination of modalities depends on the type of the seizure. ACC and EMG modalities are of added value and therefore are often used for the detection of tonic, clonic, tonic–clonic and hypermotor seizures, because substantial muscle activity and/or motion is present [4].

**Figure 1:**
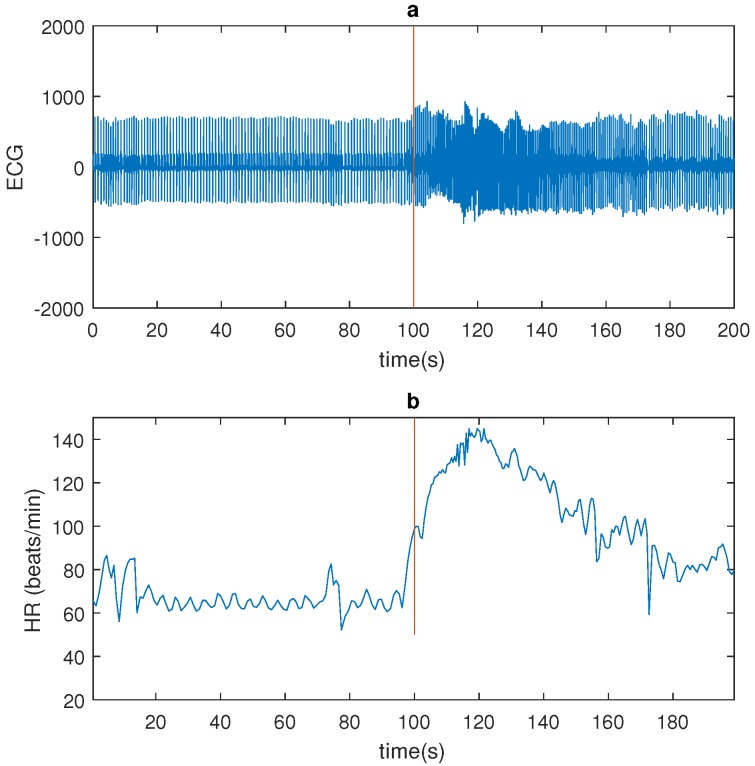
An example of a seizure (patient 4): (**a**) Electrocardiography (ECG) signal (bandpass filtered 1–40 Hz). (**b**) Heart rate (HR; red line indicates seizure onset as market by a clinician on the basis of electroencephalography (EEG)).

Temporal lobe epilepsy (TLE) seizures do not often have a motor component worth mentioning. They do however affect the autonomic nervous system, in particular the cardiovascular system. It was previously shown that temporal lobe seizures are often accompanied with a strong ictal heart rate (HR) increase [5,6,7,8]. Figure 1 shows a seizure example.

Most of the published articles about cardiac changes in epilepsy use two wired ECG electrodes, recorded together with the EEG [6,7,9,10,11,12,13]. However a wearable solution is preferred. In [14,15] wearable ECG devices are used for seizure detection. These studies focused on nocturnal epileptic seizures only, and in only a few patients, three and five respectively. To the best of our knowledge, this is the first study that compares the seizure detection performance of a wearable ECG device with the standard wired electrodes in a hospital setting during day and night.

ECG electrodes can be uncomfortable and can cause skin irritation after a few days. Another way to measure HR is by using the photoplethysmography (PPG) sensor in a smartwatch. PPG makes use of reflected light to measure changes in light absorption, caused by changes in the blood volume due to heart beats [16]. In [17], a PPG device is used, tested on two seizures only. To the best of our knowledge, no other publications exist for which the performance of a PPG device is evaluated on a multipatient dataset.

The aim of this study is to compare the performance of a wearable ECG and PPG device with the standard wired ECG. The testing was performed in a hospital setting in order to compare with the standard wired ECG and to obtain accurate seizure annotations on the basis of video-EEG. The seizure detection performances are evaluated on the same clinical database of TLE patients using an existing computer-based, automated algorithm. In a later study, the wearables, which are performing as accurately as the hospital ECG, will be tested in a real-world scenario.

## 2. Data Acquisition

The epilepsy patients were recorded with 10–20 scalp EEG with 1 bipolar ECG channel in the hospital. The two ECG electrodes were placed supraclavicularly left and right. Additionally to the standard clinical equipment, recordings were made with a wearable ECG device, the $180^{\circ }$ eMotion Faros [18], and a wrist-worn PPG device, the Empatica E4 smartwatch [19]. The Faros device was used in a one-channel configuration. The positive electrode was placed on the left side of the torso, medial-supraclavicularly; the negative electrode was placed on the left side, laterally under the rib cage. The Empatica E4 measures reflective PPG using a green and a red light-emitting diode (LED) on the wrist. The device returns a single channel, which is obtained by combining the green and the red channels using Empatica’s proprietary algorithm. The intravenous insertion, which is a needle inserted into a peripheral vein, used for administering fluids or medication, is mostly placed at the dominant wrist. As a result, the Empatica watch was worn on the other wrist. The respective sampling rates of the hospital ECG, Faros ECG and Empatica PPG signals were 250, 500 and 64 Hz. The data was managed using the Byteflies’ cloud. This system allowed for the integration of the data from the wearable devices with the hospital’s recording setup.

The dataset consists of recordings of refractory epilepsy patients, who underwent presurgical evaluation at UZ Leuven Gasthuisberg. The patients were recorded over 3, 4 or 5 days. Occasionally, due to practical or technical reasons, interruptions were present in the dataset; for example, when the patient needed to go to a scanner or when technical errors occurred. If one of the recordings was interrupted, the data of that specific time period was not analyzed. In this way, the three recording systems were evaluated exactly on the same data. Eleven patients were included. In total, 47 seizures were recorded during 701 h of data. All the seizures were complex partial and originated from the (fronto-)temporal lobe. However, the ictal EEG of patient 8 was unreadable due to muscle artifacts. Consequently, the type and origin could not be determined for that patient. A clinical expert annotated the seizure onsets and ends on the basis of the video-EEG data, without considering the ECG changes. Afterwards, the annotations were validated by a medical doctor. Table 1 gives a detailed overview of the seizure dataset. For every patient, the number of occurring seizures, the recording duration (RD), the hemisphere and origin of the epileptic focus, the age, the gender, and the mean and range of the seizure duration (SD) are given. During some seizures, the end of the seizure could not be determined, which was the case for patients 13, 17 and 23 for one seizure and for patient 6 in three seizures. The study was approved by the ethical commission of the University Hospital, Leuven. All patients gave informed consent for their participation in this study.

## 3. Methodology

The recorded datasets were analyzed using an automated algorithm implemented in Matlab. The algorithm consists of two steps: the heart/pulse rate variability (HRV/PRV) calculation and a seizure detection algorithm. The HRV quantifies the beat-to-beat variation in the HR, whereas the PRV quantifies the beat-to-beat variation in blood pulsation. Lastly, the evaluation criteria are explained.

### 3.1. HRV/PRV Extraction from ECG/PPG

#### 3.1.1. ECG

In order to extract the HRV from the ECG, a method developed in [20] was used. In the first step, the ECG signal is segmented into epochs of 60 s. Thereafter, the power line interference at 50 Hz is removed by a notch-filter, and the mean of the signal is removed. An adapted Pan-Tomkins algorithm is used to find the R-peak locations. Instead of the classical filtering steps (bandpass, derivative, and integrator), the upper ($U_{ecg}$) and lower ($L_{ecg}$) ECG envelopes are calculated. A flattened version of the ECG is defined as $F_{ecg}=U_{ecg}-L_{ecg}$, which is used to find the possible locations of the R-peaks. The HRV is calculated from the differences between the locations of the R-peaks.

#### 3.1.2. PPG

In order to extract the PRV from the PPG, a method developed in [21] was used. This algorithm consists of two phases: a linear filtering transformation and an adaptive thresholding operation. The filtering step consists of a linear-phase finite impulse response (FIR) low-pass-differentiator filter, which is used to accentuate the abrupt upslopes of the PPG pulses. The abrupt upslopes correspond to peaks in the filtered signal, which are detected by an adaptive thresholding operation. Once the peaks in the filtered signal are found, the maximum point in the original PPG signal is found. For the calculation of the PRV, the medium-amplitude point, defined as that in which the amplitude has reached 50% of its maximum, is calculated. This medium-amplitude point is shown to result in the most precise PRV. The PRV is calculated from the differences between the locations of the medium-amplitude points.

### 3.2. Seizure Detection Algorithm

In order to perform seizure detection, an algorithm developed in [6] was used. The algorithm uses the HRV or PRV as input. After filtering, the algorithm looks online for a HR increase. If this HR increase satisfies the preimposed rules [6], features are extracted and classification is performed. Three features are extracted for classification: $HR_{peak}$, ${\bar{HR}}_{base}$ and the $STDHR_{base}$. $HR_{peak}$ is the peak HR at the end of the HR increase. ${\bar{HR}}_{base}$ is the average HR over the 60 s before the start of the HR increase. $STDHR_{base}$ is the standard deviation of the HR over the 60 s before the start of the HR increase. The classification is performed with a support vector machine (SVM) classifier with a Gaussian kernel. The SVM model was trained with an independent dataset consisting of 17 TLE patients previously recorded at UZ Gasthuisberg [6].

### 3.3. Evaluation Criteria

Four metrics were used to describe the seizure detection performance. The sensitivity (Se) and false positives per hour (FP/h) were calculated. Seizures were detected when an alarm was given between 30 s before and 90 s after the seizure onset. False alarms within 1 min of each other were counted as one alarm. Additionally, the positive predictive value (PPV) was calculated. Average measures over the entire dataset can be expressed as a patient-average performance (Pat.-av.) (the average of the performance of each patient) or an overall performance (Tot.-av.) (computed on the total number of seizures or recording duration). The latter was used, unless specifically mentioned. Lastly, the sensitivity and false alarm rate were calculated for different thresholds, similarly to a receiver operating characteristic (ROC) curve. In this context, it is more meaningful to plot sensitivity against FA/h instead of the classical true positive rate against the false positive rate. It is informative to know how the sensitivity is affected by the seizure duration. Therefore the seizure sensitivity is calculated as a function of the seizure duration, which is calculated using the start and end of the EEG seizure pattern, annotated by a medical doctor.

### 3.4. Evaluation of Sensitivity and False Alarms

In order to compare the different modalities in detail, the underlying cause of missed seizures and false alarms should be investigated. The reasons for the missed seizures and false alarms may be physiological or related to the data quality of the recording system. A first possible physiological reason is that no HR changes are observed during the seizure. Second, the HR increase may be too small (lower than 20 beats per minute) to be detected by the algorithm. Third, a HR decrease may be observed during a seizure. Quality-related reasons in this dataset were motion artifacts and interference. A physiological reason for false alarms is the occurrence of a HR increase during a non-ictal period, for example, during light physical exercise. Similarly to the missed seizures, a quality-related reason is the presence of motion artifacts. Furthermore, it is interesting to know if these false alarms of the different modalities occur at the same time. For every patient, the percentage of coincidences of the FPs were calculated for the different modalities.

## 4. Results

**Figure 2:**
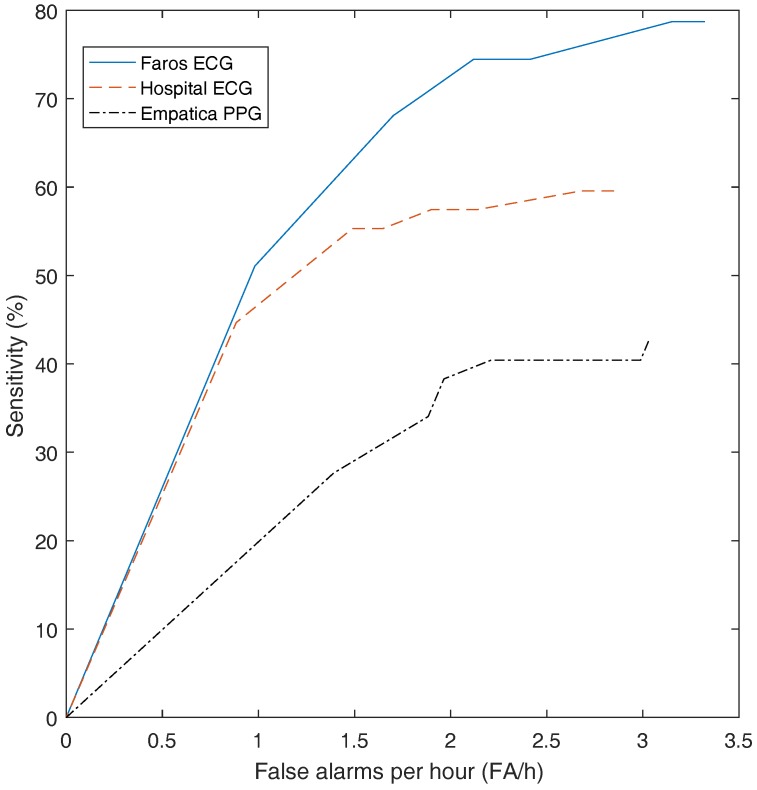
The sensitivity and false alarm rate for different thresholds of the support vector machine (SVM) classifier.

**Figure 3:**
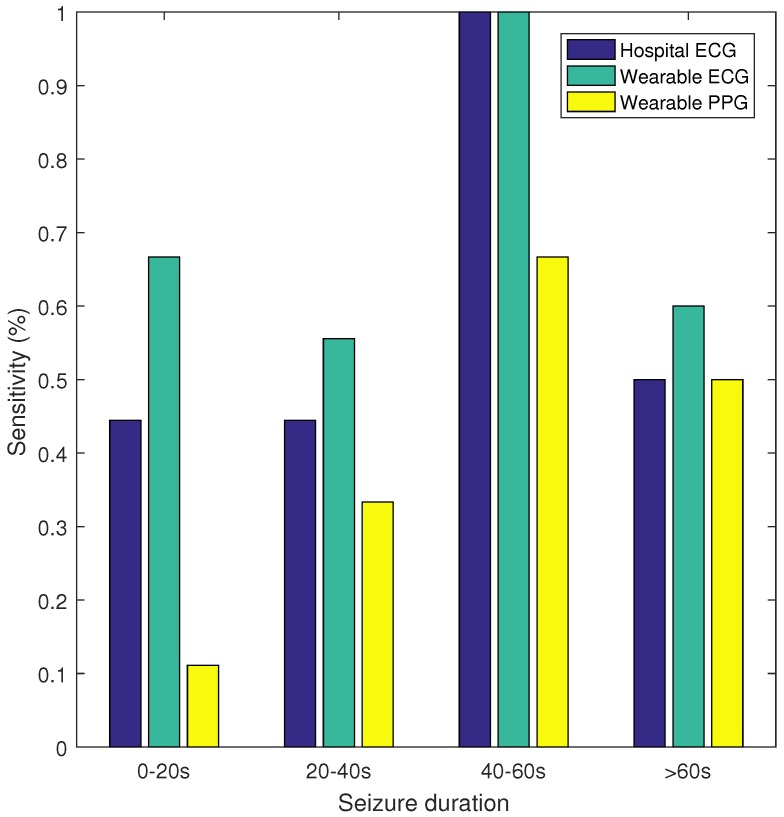
Sensitivity versus seizure duration.

Table 2 gives an overview of the results. The highest sensitivity (70%) was obtained with the wearable ECG, whereas the sensitivity of the hospital ECG was 57%. With the wearable PPG, a sensitivity of only 32% was obtained. The FP/h values are comparable for the three recording systems: 1.92, 2.11 and 1.80 for the hospital ECG, the wearable ECG and the wearable PPG, respectively. In Figure 2, the sensitivity and false alarm rate for different thresholds of the SVM classifier are plotted. This curve shows that for the same FP/h value, the wearable ECG achieved a higher sensitivity than the hospital ECG, whereas the wearable PPG had a lower sensitivity. Additionally, sensitivity was calculated for seizures of different durations, namely, 0–20, 20–40, 40–60, and >60 s. In Figure 3 the sensitivity is plotted for the hospital ECG, the wearable ECG and the wearable PPG for the different seizure durations. The seizures with an unknown seizure duration were neglected by this analysis. Unfortunately, the number of seizures in each of the seizure length categories (9, 9, 3 and 10) were too small to draw conclusions.

### 4.1. Sensitivity

**Figure 4:**
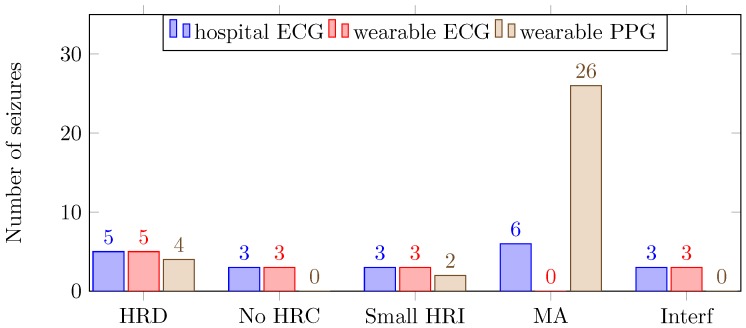
Number of and reason for missed seizures (HRD: heart rate decrease; No HRC: no heart rate change; Small HRI: small heart rate increase: MA: notion artifacts; Interf: interference).

The number of missed seizures for the hospital ECG, the wearable ECG and the wearable PPG were 20, 14 and 32, respectively. In Figure 4, the number of and reason for missed seizures are shown for the three different recording systems.

### 4.2. False Alarms

The obtained false alarm rates were in accordance with previously published results [6]. The average (± standard deviation) FP coinciding from the wearable ECG and wearable PPG with respect to the hospital ECG were respectively 63%±20% and 35%±16%. The FP coincidence of the wearable ECG with respect to wearable PPG was 36%±14%.

## 5. Discussion

In order to have a good seizure detection performance, the sensitivity should be as high as possible, whereas the false alarm rate should be as low as possible. A statistical evaluation of the seizure detection performance is very important [22,23]. It has been proven previously [6] that the applied seizure detection algorithm performs better than chance. Here, using wearable ECG, we obtained a comparable seizure detection performance to that of [6]. Below, we discuss in detail the reasons for missed seizures and false alarms.

### 5.1. Sensitivity

In the dataset, 11 seizures could not be detected because of physiological reasons: HR decrease (5), no HR changes (3) and a small HR increase (3). Logically, these seizures appeared as missed seizures, with the exception of PPG signals, for which five of the seizures were detected because of motion artifacts. In other words, in this dataset, 77% of the seizures were accompanied with a HR increase. In previous research, 88% and 92% of the temporal lobe seizures were accompanied with a HR increase [8,11]. Furthermore, the same seizure detection algorithm obtained a sensitivity of 82% for another dataset of temporal lobe seizures [6]. Adding HR-based information is only suitable for patients who have a change in cardiac activity during a seizure. For this dataset, patients 1, 2 and 9 did not show a strong HR increase. In order to increase sensitivity, a prescreening is useful to determine if ECG is a suitable modality for the patient.

Both for the hospital ECG and the wearable ECG, three missed seizures were due to the presence of interference, which was around 12 Hz. This interference was difficult to remove because the R-peaks of the ECG signals contained that particular frequency. Fortunately, this was only present during one day of recording, for patients 8 and 10. In the rest of the dataset, this was not observed. It is likely that there was an electronic device present close to the ECG equipment. Because the PPG does not pick up an electrical signal, no interferences were observed in the PPG.

**Figure 5:**
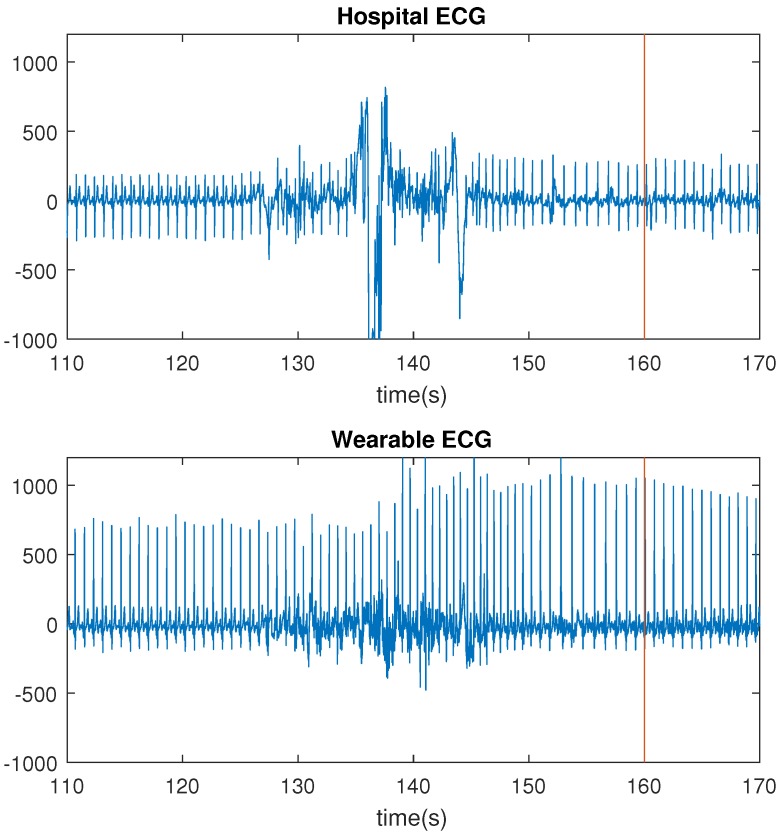
Comparison of motion artifacts: hospital electrocardiography (ECG) and wearable ECG (red line indicates annotated seizure start).

The number of missed seizures, as a result of motion artifacts, differred greatly for the three recording systems. For the hospital ECG, the wearable ECG and the wearable PPG, the numbers were respectively 6, 0 and 26. The wearable ECG performed better than the hospital ECG because of the lack of wires. Motion affects the wires of the hospital ECG, thereby including small displacements of the electrodes, resulting in motion artifacts in the signals. In Figure 5, the HR increase, corresponding to a seizure of patient 2, is shown. For this seizure, the HR increase occurred before the seizure onset. Both ECG signals were bandpass filtered between 1 and 40 Hz with a butterworth filter.

The PPG signal was drastically affected by the motion artifacts; 55% of the seizures could not be detected because of motion artifacts, which is unacceptably high. These motion artifacts are caused by (1) the movement of venous blood as well as other non-pulsatile components, and (2) variations in the optical coupling between the sensor and the skin [24,25]. As a result of these motion artifacts, no reliable HR could be extracted by the algorithm. Furthermore, by visually inspecting the power spectra, the HR frequency was often no longer present in the signal.

The PPG wristband is mostly worn on the dominant hand, because the intravenous insertion is typically performed on the non-dominant hand. The dominant hand is expected to move even more compared to the non-dominant hand; therefore, in the home environment, placing the watch on the non-dominant hand may bring about some improvement. Alternatively, the smartwatch can be placed on the upper arm [17]. Further research is needed to investigate positions with which to measure the PPG.

### 5.2. False Alarms

The coincidences of FPs for the hospital and wearable ECG were substantially greater than for the wearable PPG. It is expected that the FPs with physiological causes will coincide. In contrast, the FPs due to quality are specific for each recording system. Therefore we speculate that the FPs of the wearable PPG are more often due to motion artifacts.

### 5.3. Limitations and Further Work

In this work, the quality of a wearable ECG and PPG device are compared against the hospital ECG. The comparison was performed by running the same seizure detection algorithm on different datasets. However, one can also compare the quality of the signals or the extracted HR, which is more direct. By doing so, one ignores the fact that the algorithm can cope with certain artifacts and wrong HR estimates. Yet, we chose this approach as we are also interested in how the seizure performance is affected by such artifacts.

Another limitation is that the seizure classifier is trained with the HR extracted from an ECG signal and not from a PPG signal. This might cause some bias, because the nature of artifacts is different and will have a different influence on the seizure classifier performance.

The recordings were taken in a hospital environment, where the patient was limited in their natural daily behaviour because of the wired hospital recording system. In a daily life or home environment, there may be even more artifacts present.

Noise removal algorithms should be tested on the PPG data to reconstruct the HRV information [26].

In this work, only data of (fronto-)TLE patients is analyzed. The seizure performance of other patient groups is not evaluated.

The false alarms rate per hour for the three detection systems were around 2 FP/h (Table 2), which is comparable with those in the literature [6]. A comparison between different state-of-the art HR-based independent detection algorithms is included in [6]. These algorithms have on average two false alarms per hour, which is not acceptable for practical use. In order to reduce false alarms, a patient-dependent or adaptive algorithm should be considered. An algorithm with tunable parameters and thresholds is provided in [14,15]. In this case, medical data should be available for that patient. In [27], an online algorithm is developed, which starts from a patient-independent algorithm and adapts to the characteristics of the seizures of that specific patient. For this, no data of the patient is needed beforehand. Another way to reduce false alarms is the combination of multiple signals. Depending on the seizure characteristics, other biomedical signals can be integrated [4]. However the aim of this paper is to compare the performance of an ECG and a PPG wearable device with the ECG hospital system, not to outperform the state-of-the-art seizure detection systems.

## 6. Conclusions

The performances of two wearable devices (ECG and PPG) are compared with the hospital ECG using an existing automated algorithm based on cardiac activity. The algorithm was tested on a dataset of 11 patients in a hospital setting over 701 h and 47 (fronto-)temporal lobe seizures. Whereas seizure detection performance using the wrist-worn PPG device was considerably lower, the performance using the wearable ECG is proven to be similar to that of the hospital ECG. This suggests that the wearable ECG has potential to be used for long-term monitoring. Future work will further evaluate the signal quality and seizure detection performance in daily life outside the hospital.

## Figures and Tables

**Table 1:** An overview of the dataset, RD = Recording Duration, SD = Seizure Duration.

Patient	Seizures	RD (h)	Hemisphere	Origin	Age	Gender	Mean SD (s)	Range SD (s)
1	6	20	Left	Temp	28	M	28.7	[26 31]
2	3	72	Left	Temp	49	F	51	[24 68]
3	1	72	Bihemi	Temp	67	F	26	[26 26]
4	7	73	Right	Fronto-temp	24	F	47.5	[33 85]
5	8	95	Right	Temp	32	M	46	[25 61]
6	2	23	Left	Temp	19	F	83	[83 83]
7	1	43	Left	Temp	64	M	55	[55 55]
8	10	71	EEG not readable		49	M	17	[8 30]
9	2	91	Right	Temp	61	M	250	[34 466]
10	5	71	Left	Temp	45	M	63.8	[11 95]
11	2	70	Left	temp	32	F	43.5	[20 67]
Total	47	701						

**Table 2:** Seizure performance: The sensitivities (Se), false positives per hour (FP/h) and positive predictive value (PPV) are shown with the patient average (Pat.-av.) and total average (Tot.-av.).

Patient	Hospital ECG			Wearable ECG			Wearable PPG		
	Se (%)	FP/h	PPV (%)	Se (%)	FP/h	PPV (%)	Se (%)	FP/h	PPV (%)
1	33	3.98	2.50	33	3.98	2.44	33	1.77	5.26
2	0	1.55	0.00	33	2.06	0.67	33	1.49	0.92
3	100	1.55	0.89	100	2.09	0.65	0	2.27	0.00
4	71	2.27	2.94	100	2.89	3.23	43	1.91	2.11
5	88	2.30	3.10	88	1.96	3.61	38	1.55	1.99
6	50	2.91	1.02	50	4.24	0.99	100	2.41	3.39
7	100	0.45	5.00	100	1.03	2.22	0	1.64	0.00
8	50	3.44	2.02	90	2.64	4.62	20	2.53	1.09
9	0	0.40	0.00	0	0.62	0.00	0	1.08	0.00
10	80	1.03	6.56	75	1.53	3.54	0	2.06	0.00
11	50	2.73	0.34	50	2.78	0.36	100	1.94	1.04
Pat.-av.	57	2.05	2.22	64	2.35	2.03	33	1.88	1.43
Tot.-av.	57	1.92	1.93	70	2.11	2.15	32	1.80	1.12

## Abbreviations

The following abbreviations are used in this manuscript:

EEG	Electroencephalography
ACC	Accelerometry
EMG	Electromyography
ECG	Electrocardiography
TLE	Temporal lobe epilepsy
HR	Heart rate
PPG	Photoplethysmography
RD	Record duration
SD	Seizure duration
HRV	Heart rate variability
PRV	Pulse rate variability
FIR	Finite impulse response
SVM	Support vector machine
Se	Sensitivity
FP	False positives
PPV	Positive predictive value
ROC	Receiver operating characteristic
LED	Light emitting diode
Pat.-av.	Patient average
Tot.-av.	Total average
